# PI3Kα-RAS interface disruption as a new therapeutic strategy

**DOI:** 10.1016/j.gendis.2025.101905

**Published:** 2025-10-27

**Authors:** Abdelhalim Azzi

**Affiliations:** Laboratory of Lipids and Chronobiology, IMol, Polish Academy of Sciences, Warsaw 02-247, Poland

The phosphoinositide 3-kinase (PI3K) family and the PI3K/AKT signaling pathway have been recognized as key players in oncogenic processes such as cell survival, metabolism, and metastasis. PI3K transduces signals from growth factors into intracellular responses by converting PI (4,5) P2 into PI (3,4,5) P3, activating the serine–threonine protein kinase AKT and downstream pathways. The PI3K/AKT pathway is tightly regulated, but in cancer, it can be constitutively activated through mutations in PI3K enzymes or receptor tyrosine kinases, such as EGFR or HER2. An alternative mechanism for activating the PI3K/AKT pathway involves the direct interaction between PI3K and the RAS protein. This interaction facilitates the recruitment of PI3K to the plasma membrane, promoting its catalytic activity.^1^ Disruption of the RAS-PI3Kα interaction alters tumour formation, highlighting its role in tumourigenesis.^2^ Based on the role of the PI3K/AKT pathway in cell growth and proliferation, it has been recognized as an attractive target for treating cancer. Several PI3K inhibitors have been characterized, some of which have already been approved for therapeutic use.^1^ However, most of these inhibitors are primarily ATP-competitive, which limits their specificity. While effective in suppressing tumor growth, their systemic application often causes hyperglycemia and insulin feedback by disrupting normal PI3K activity, limiting their tolerability and long-term clinical use^3^ (Fig. 1A). In a recent study, Simanshu DK et al^4^ presented an alternative approach by targeting the protein–protein interaction between RAS and the Ras-binding domain (RBD) of PI3Kα, thereby decoupling oncogenic activation from essential metabolic signaling.

**Figure 1:**
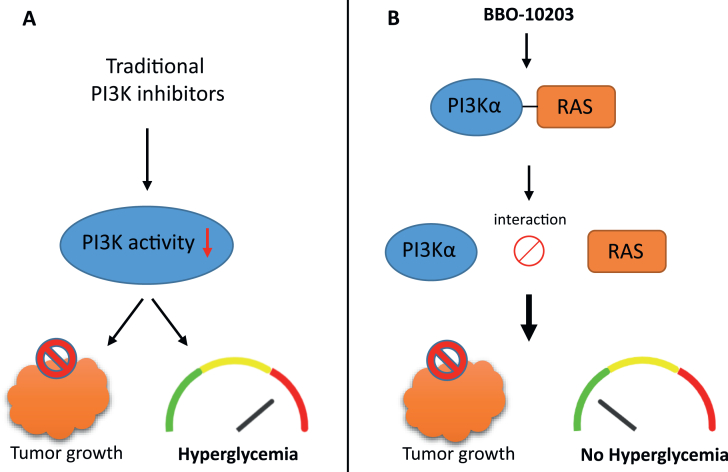
Comparison of the effects of traditional PI3Kα inhibitor and BBO-10203 on tumor growth and glucose regulation. (**A**) Traditional PI3Kα inhibitors block PI3Kα signaling and inhibit tumor growth but also induce hyperglycemia. (**B**) BBO-10203 selectively targets the PI3Kα-RAS interaction without interfering with insulin signaling, thereby inhibiting tumor growth while preventing hyperglycemia.

## BBO-10203 selectively inhibits RAS-mediated PI3Kα activation

Through structure-based drug design and molecular modelling, BBO-10203 was identified as a small molecule that binds to the RBD of PI3Kα and interferes with its association with RAS. This interaction is specific to PI3Kα but not to other PI3K isoforms. The inhibition of PI3Kα was validated through biochemical assays, including mass spectrometry, isothermal titration calorimetry, and co-immunoprecipitation, confirming the high specificity for the RAS–PI3Kα interaction.^4^ Compared to Alpelisib, a known ATP competitive inhibitor of PI3Kα, BBO-10203 did not inhibit ATP binding or lipid kinase activity of this enzyme. Interestingly, BBO-10203 markedly reduced AKT phosphorylation, with a stronger effect on HER2-amplified cells, which are highly dependent on PI3Kα signaling. In KRAS-mutant cells, BBO-10203 revealed wide variability in AKT inhibition, suggesting that AKT phosphorylation is not entirely dependent on active RAS, indicating the existence of alternative activation routes. *In vivo*, BBO-10203 effectively inhibited AKT phosphorylation and suppressed tumor growth in an esophageal cancer model. Furthermore, combining BBO-10203 with other therapeutic agents, such as the CDK4/6 inhibitor, trastuzumab, or the KRAS-G12C inhibitor BBO-8520, resulted in marked tumour regression.^4^ These findings suggest that BB1230 could be a game changer in PI3K-targeted cancer therapy.

## Preserving metabolic function while targeting tumor growth

In addition to its antitumor activity, BBO-10203 did not induce hyperglycemia in treated models, highlighting a key safety advantage over other PI3Kα catalytic inhibitors, which are often limited by dose-limiting their metabolic toxicities. BBO-10203-treated mice maintained normal fasting glucose levels and did not show signs of hyperglycemia during glucose tolerance testing or hyperinsulinemia.^4^ These findings demonstrate that the physiological PI3Kα activity required for maintaining systemic glucose homeostasis remains intact. The underlying mechanism is that insulin and growth factor signaling activate PI3Kα via distinct inputs, whereas RAS activates PI3Kα through direct binding to its RBD. The findings reported in this study suggest that BBO-10203 interrupts only the latter pathway, selectively targeting the tumor-promoting activity while preserving normal signaling in metabolic tissues (Fig. 1B).

## Mechanistic insight into interface disruption

The Ras-binding domain of PI3Kα, located on the surface of the p110α catalytic subunit, mediates direct binding to active RAS proteins. BBO 10203 functions by targeting the RBD. Structural analysis and modeling of the BBO-10203-PI3Kα interaction involve key amino acid residues adjacent to the region known to be in critical contact with RAS.^4^ Point mutations in this region abrogate RAS binding and selectively impair oncogenic PI3K signaling while sparing basal kinase activity, which is similar to the mechanism observed for BBO-10203.^2^^,^^4^ The RAS–PI3Kα interaction has been well-characterized, and membrane-resident HRas recruits PI3Kα to the membrane, leading to changes in its structure and promoting its lipid kinase activity.^5^ Disruption of this interaction alters growth factor signalling and inhibits cellular transformation.^3^ Interestingly, this RAS-PI3Kα interaction is not required for insulin signalling.^5^ Therefore, the RBD region of class I PI3K is an ideal point of intervention to specifically and selectively suppress oncogenic signaling driven by RAS on PI3Kα. Moreover, given the differences in amino acid sequences among the three class I PI3K isoforms within the RBD, one could speculate that other inhibitors selectively targeting each isoform in this region could be developed more easily using a strategy similar to the one described in this study. Although challenging, the strategy of targeting protein–protein interactions has broader implications in cancer treatment. BBO-10203 is another member of this emerging class of allosteric interface inhibitors, demonstrating that region-specific targeting can achieve high therapeutic precision.

## Implications for future therapeutic strategies

Inhibitors of small molecules targeting PI3K, including Alpelisib (approved for PIK3CA-mutant breast cancer), idelalisib, and copanlisib, have demonstrated on-target efficacy but can cause adverse effects such as hyperglycemia.^1^^,^^3^^,^^4^ These side effects are mainly attributed to the disruption of the PI3K signaling in metabolic tissues such as the liver and muscle, where PI3Kα plays a central role in insulin-mediated glucose uptake. BBO-10203 overcomes this limitation, preserves PI3Kα activity and does not induce hyperglycemia. Moreover, the preclinical data presented in this study suggest strong potential for combination strategies, further supporting the therapeutic potential of targeting the RAS-PI3Kα protein interaction. This new approach represents a major shift in PI3K-targeted therapy, opening avenues for addressing the persistent challenge of dose-limiting toxicities and the limited effectiveness seen with pan- and isoform-selective PI3K inhibitors. Furthermore, this protein–protein interaction targeting strategy could also be applied to the development of inhibitors against other class I PI3K isoforms, as well as phosphoinositide phosphatases such as PTEN and SHIP2, potentially enabling more precise modulation of phosphoinositide signaling with reduced off-target effects.

## Declarations

The AI tools Grammarly and ChatGPT were used only at the final stage of the manuscript preparation and solely for English corrections and clarifications.

## CRediT authorship contribution statement

**Abdelhalim Azzi:** Writing – review & editing, Writing – original draft, Funding acquisition, Conceptualization.

## Funding

This work was supported by the 10.13039/501100004442National Science Centre, Poland, NCN SONATA BIS grants to A. AZZI (No. 2022/46/E/NZ3/00144).

## Conflict of interests

The author declares no competing interests.
